# Obesity and renal disease: Benefits of bariatric surgery

**DOI:** 10.3389/fmed.2023.1134644

**Published:** 2023-02-28

**Authors:** Leopoldo G. Ardiles

**Affiliations:** Department of Nephrology, Faculty of Medicine, Universidad Austral de Chile, Valdivia, Chile

**Keywords:** obesity management, metabolic syndrome, chronic kidney disease, bariatric surgery, overweight

## Abstract

The prevalence of obesity, a preventable and reversible condition with a high impact on health, continues to rise, especially after the COVID-19 pandemic. Severe overweight is well recognized as a risk factor for diabetes and hypertension, among other conditions, that may increase cardiovascular risk. Obesity has grown simultaneously with a rise in the prevalence of chronic kidney disease, and a pathophysiological link has been established, which explains its role in generating the conditions to facilitate the emergence and maximize the impact of the risk factors of chronic kidney disease and its progression to more advanced stages. Knowing the mechanisms involved and having different tools to reverse the overweight and its consequences, bariatric surgery has arisen as a useful and efficient method, complementary or alternative to others, such as lifestyle changes and/or pharmacotherapy. In a detailed review, the mechanisms involved in the renal consequences of obesity, the impact on risk factors, and the potential benefit of bariatric surgery at different stages of the disease and its progression are exposed and analyzed. Although the observational evidence supports the value of bariatric surgery as a renoprotective measure in individuals with obesity, diabetic or not, randomized studies are expected to establish evidence-based recommendations that demonstrate its positive risk–benefit balance as a complementary or alternative therapeutic tool.

## Introduction

The continuous growth in the prevalence of overweight, aggravated by the COVID pandemic (1), is causing metabolic, orthopedic, psychological, and cardiovascular consequences and revealing other relevant damages with clinical significance (2).

The prevalence of obesity, age-standardized, among adults aged 18 and older, defined as a body mass index (BMI) of >30 kg/m^2^, has been rising over the past few decades, reaching a worrying figure of 650 million obese adults in the world in 2016 (3). Obesity is prevalent, especially in first-world countries where it continues to increase (4). Between 2005 and 2015, the obesity rate was approximately 32% in the United States and 24% in the United Kingdom (5). Data from the Framingham cohort show that not only the degree of obesity but also the period of exposure is important (6). Obesity is not only associated with increased mortality but also is a main risk factor for many diseases including chronic kidney disease (CKD) (7), nephrolithiasis, and renal cell cancer (8) (Table 1).

**Table 1:** Clinical consequences of obesity in the kidney.

Fatty kidney
Salt-sensitivity
Albuminuria and proteinuria (sub-nephrotic levels)
Hyperfiltration and progressive chronic kidney disease
Increase in the risk of progression of non-obese-related nephropathies
Indirect effects mediated by sleep-apnea and nocturnal hypoxemia and non-alcoholic fatty liver disease
Nephrolithiasis

In addition, approximately 15% of United States adults (37 million people) are estimated to have CKD, with diabetes and hypertension being the most common causes. Other risk factors such as cardiac disease, family history of CKD, inherited kidney disorders, previous renal injuries, older age, and obesity are now recognized (9). In the National Health and Nutrition Examination Survey, 44% of patients with CKD in the United States were obese (22% with class 1 obesity, 11% with class 2, and 11% with class 3), with an overall percentage increasing through the following years. This prevalence is higher than that in the general population (36%) (10). In addition, CKD is the second leading cause of death-and disability-adjusted life years (11), and data show that more than two-thirds of deaths related to obesity are due to cardiovascular disease (CVD) in the world (12).

Efforts to reduce the systemic, particularly the renal, effects of the overweight pandemic must be adopted seriously, using the best available tools, especially if the knowledge about the effects of obesity on the kidney and the benefits of its reversal is growing. Bariatric surgery techniques are emerging as the most successful weight loss strategies for adults with extreme obesity, and their application in patients with chronic kidney disease deserves to be validated.

## Epidemiological and pathophysiological bases

A definitive link between obesity and renal damage was established in 1923 by Preble (13), increasing in a stepwise fashion as BMI rises, even adjusted by the existence of hypertension, diabetes, history of smoking, and CVD (14). Indeed, obese individuals had a 3-fold higher risk of CKD than normal-weight healthy subjects (14, 15), regardless of their metabolic status and even in the absence of remarkable metabolic abnormalities (16). The most recognized link between obesity and CKD is centered on the insulin resistance associated with glucose intolerance, dyslipidemia, and hypertension that frequently progresses to overt type 2 diabetes mellitus (T2DM) (17). The relationship between diabetes and obesity is so close that it has led to the coining of the term “diabesity” (18). The relevance of obesity and its impact on the incidence and progression of CKD deserves attention because of its high prevalence, broad impact on health outcomes, and its modifiable nature (19).

A big meta-analysis of cohort studies, including 600,000 cases, assessed the effect of obesity on the CKD risk beyond its association with T2DM: obesity could be associated with a 51% increase in the risk of new-onset albuminuria and 18% in the risk of new onset of CKD stage 3 at 5 years (20). Furthermore, an Israeli army study reported hazard ratios for kidney failure of 3 and 6.9 for individuals with overweight and obesity, respectively, compared to normal BMI subjects. It is interesting to observe that having overweight (BMI between 25 and 29.9 kg/m^2^) could increase or not the risk (6, 14), suggesting a weight threshold.

Although diabetes and hypertension alone explain most obesity-associated renal risk, in many observational studies, obesity is independently associated with the development of proteinuria, acute kidney injury, CKD, and end-stage kidney disease, both in otherwise healthy and in higher-risk groups like prehypertensive individuals (21–24). In addition, obesity may accelerate the loss of function in a variety of renal diseases such as polycystic disease, IgA nephropathy, renal transplant, and diabetic renal disease (25–27). Furthermore, low birth weight, low renal endowment, or any cause of reduced renal mass display an increased risk of progression to CKD and end-stage kidney disease (ESKD) when associated with obesity (28).

### Different compartments in the kidney may be affected by obesity

#### Glomerular lesions

Obesity-associated glomerulopathy has been described in obese individuals with a BMI of >30 without clinical or histopathological evidence of other diseases and is clinically manifested by proteinuria (2, 29). Patients with obesity show glomerular hypertrophy associated with low glomerular density with or without a characteristic form of focal segmental glomerulosclerosis (FSGS), increased renal plasma flow (RPF), and glomerular filtration rate (GFR). In adaption to the glomerular expansion, podocyte increase in size but cannot keep up with it, leading to podocyte failure and detachment that results in an increase in glomerular permeability followed by lesions of FSGS (30).

#### Tubular lesions

Proximal tubular hypertrophy, with a higher cross-sectional area and its lumen, has been described in proteinuric obese individuals compared to non-obese patients with proteinuria (31) and may be related to the hyperfiltration state like the one observed in diabetes (32). Animal models have shown lipid cytoplasmic inclusions, tubulointerstitial inflammation, and fibrosis reversed by weight loss (33).

### Direct and indirect effects of obesity on the kidney

Adipose tissue is the primary location designated to accumulate the excess energy in the form of triglycerides in specialized cells called adipocytes. Fat is not only a reservoir compartment but also produces secretory factors that may affect renal function, inducing insulin resistance, RAAS (renin angiotensin aldosterone system) activation, and eventually, renal inflammation (34). Two types of adipose tissue can be recognized according to structure and function: white adipose tissue specially dedicated to storage (35) that may be widely found surrounding organs such as the kidneys, and brown adipose tissue containing uncoupling protein 1 (thermogenin) present in mammalians, which transfers energy into heat to protect survival in cold stress situations such as the birth or hibernation with low access to proteins (36). Brown fat is associated to a metabolically health obesity phenotype, less associated to insulin resistance, inflammation, hypertension or T2DM (37).

**Figure 1:**
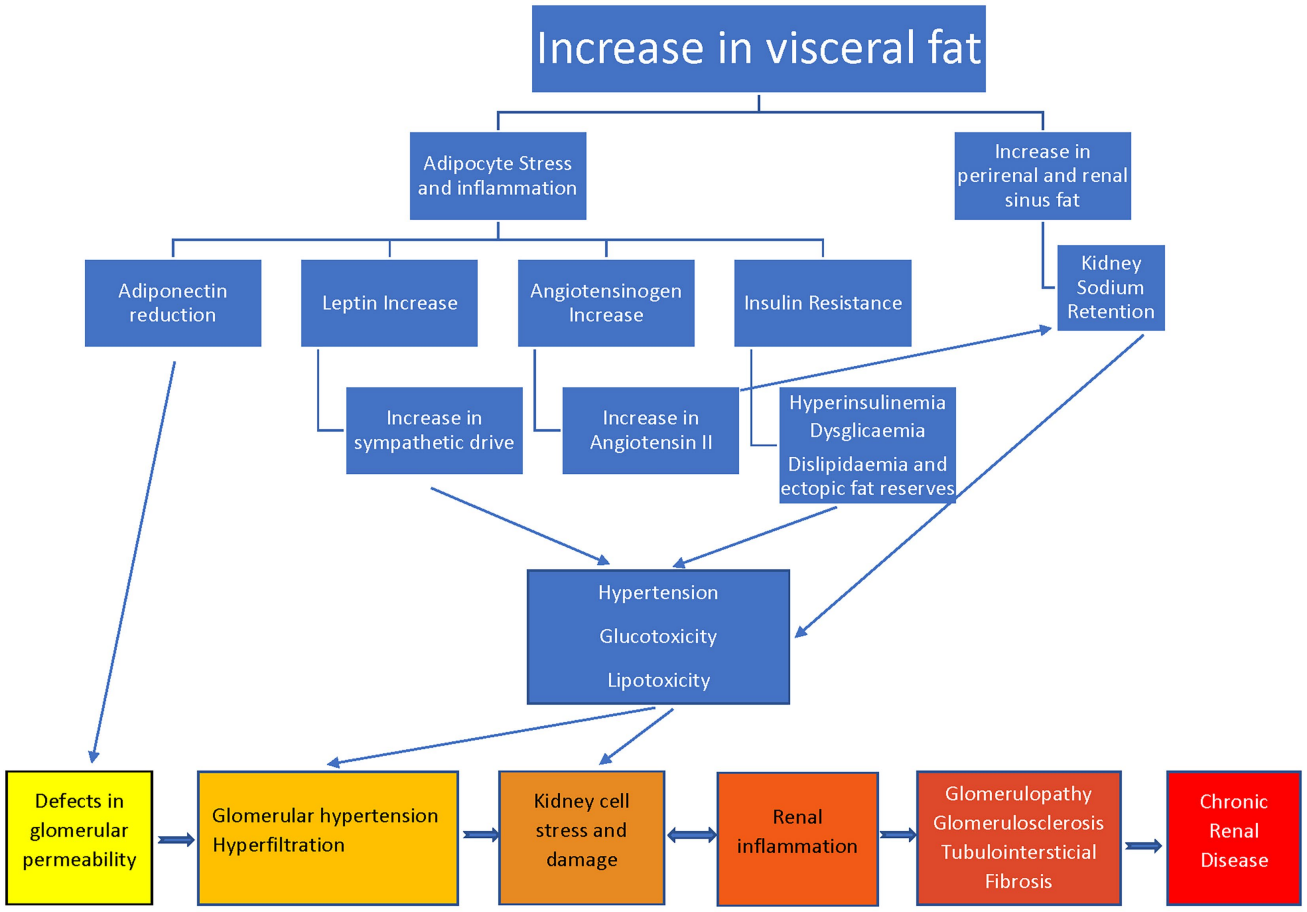
Pathophysiological mechanisms and consequences of obesity on the kidney.

Mechanisms of damage in obesity-associated kidney disease involve hemodynamic (hyperfiltration, podocyte damage, and renin–angiotensin–aldosterone system activation), metabolic (dyslipidemia and adipokine dysregulation) and lipid nephrotoxicity (renal fat deposits knowns as a fatty kidney) (38), and oxidative stress with lipid peroxidation (39) (Figure. 1).

An obese state is characterized mainly by an increase in white adipose tissue and it is associated with a low-grade systemic inflammation through an increased release of pro-inflammatory products (TNF-α and IL-6) and a reduced effect of favorable adipokines (leptin and adiponectin) (40).

The participation of inflammatory markers in the pathogenesis of glomerular hyperfiltration (a key element in the pathogenesis of kidney damage) deserves special mention since it opens a new avenue of pathophysiological studies that can provide diagnostic, prognostic, and therapeutic guidelines. There is published evidence showing that the activation of the NLRP3 inflammasome is an effect shared by obesity and insulin resistance (41). Recently, an inverse correlation has been found between insulin sensitivity and serum levels of interleukin-1beta (42); in addition, in patients undergoing bariatric surgery, the hyperfiltration reverses only in those who show a reduction in circulating levels of IL1b/caspase-1 (43).

Leptin, the physiological satiety hormone secreted by adipose tissue, inhibits insulin synthesis and secretion, decreasing energy expenditure and food intake by stimulation of hypothalamic receptors (44). Obesity has been suggested to be a leptin resistance state, where it loses its satiety properties but maintains its adrenergic effects on non-thermogenic tissues, inducing salt-retention, vasoconstriction, and hypertension, and raising the cardiovascular risk and the rate of progression of kidney diseases (45). In addition, adiponectin has anti-inflammatory properties inhibiting the production of TNF-α and IL-6, metabolic actions sensitizing the liver, fat, and muscular tissue to the action of insulin, with an active role in atherosclerosis protection (46).

Just as it occurs for proinflammatory cytokines, something similar is observed for adipokines. A decrease in leptin and visfatin levels and an increase in adiponectin can be observed after successful bariatric surgery in patients with established CKD (47).

Some authors have linked low levels of adiponectin to an increase in cardiovascular risk in obese and hypertensive individuals with renal damage (48), but others have reported an increase, arguing an adiponectin-resistant condition (49). In addition, local production of components of the RAAS, such as angiotensinogen in the adipose tissue itself, has been found to play a pathogenetic potential in hypertension and salt sensitivity frequently found in patients with obesity (50). Furthermore, an ectopic fat storage syndrome has been proposed, when the capacity of adipocytes is exceeded and dietary lipids or calories are accumulated intracellularly in non-adipose tissues inducing lipotoxicity and dysfunctions (51). These metabolic abnormalities are almost always accompanied by the presence of hyperglycemia and its well-known effects in the kidney (52). In addition, a mechanical process may be triggered by excess adipose tissue deposition in the kidney inducing compression to enhance sodium reabsorption by reducing the peritubular renal blood flow and intraglomerular hypertension–hyperfiltration by renal vein ectasia (17, 38, 53). The measurement of renal fat deposits has been proposed as a biomarker of obesity-associated renal damage, using techniques such as ultrasonography, elastography, computed tomography, and nuclear magnetic resonance (54).

With this pathophysiologic substrate, obesity may be considered an inflammatory state in the context of hypertension, insulin resistance, and high cardiovascular risk, involved in renal damage and its progression. BS is protective against renal function decline in patients with severe obesity in the long term, in coincidence with an improvement in inflammation (55). The metabolic syndrome, a constellation of central obesity, hypertension, dysglycemia, insulin resistance, atherogenic dyslipidemia, and non-alcoholic fatty liver disease, has been associated with increased CVD, T2DM, and CKD incidence. Severely obese patients with an accompanying diagnosis of diabetes carry the highest risk for CKD (56). However, the risk of CKD in obesity is beyond this association, information that was obtained in a big meta-analysis (20), showing that a high BMI in obese individuals is a significant and independent risk factor for the new onset of albuminuria and an estimated glomerular filtration rate (eGFR) of <60 mL/min/1,73 m^2^. This fact has been observed also in studies of twins with different BMI (57).

### Weight reduction and renal protection

Different strategies, invasive or not, have demonstrated efficiency in the reduction of BMI (58–61). They include changes in lifestyle, diets, pharmacological intervention as the most used (Table 2), and recently an expansion in the use of bariatric surgery (BS). No matter how, only a reduction in body weight can induce positive changes in obese patients with renal manifestations (62, 63). The first proofs of these effects were observed in a small group of patients with advanced diabetic nephropathy (64). After a very low-calorie diet for 12 weeks, a significant reduction of BMI was accompanied by improvement in glomerular filtration markers (serum creatinine, cystatin C, and eGFR) although without significant changes in the levels of albuminuria or proteinuria. In addition, reductions in fasting glucose, insulin levels, and HOMA model score were obtained. Furthermore, a long-term behavioral weight loss intervention in overweight or obese adults with T2DM (62) obtained significant weight loss, improvement in glycosylated hemoglobin levels, reduction in blood pressure, and 31% of reduction in cumulative incidence of very high-risk CKD at 8 years compared with a group with diabetes support and education.

**Table 2:** Alternatives or complementary therapies to bariatric surgery in obese patients with CKD.

Therapies	Problems	Advantages
Lifestyle modifications (diet, exercise)	Adherence in the long term	Feeling of well-being
RAAS inhibition	Beneficial effects can be depleted as some individuals gain further weight. Hyperkalemia, renal dysfunction, cough, angioedema	Oral administration, low cost
SGLT2 inhibitors	Glycosuria (some patients may experiment higher frequency of urogenital infections)	Oral administration
		Anti-inflammatory effects
		Reduce blood pressure
		Reduce glycemic levels
		Reduce hyperlipidemia
		Increase natriuresis
		Reduce renal hyperfiltration and proteinuria
		Intrinsic reno-cardio-vascular protection
Melatonin	May alter circadian cycles	Oral administration
		Improves sleep
		Antioxidant, Anti-apoptotic
		Modules sympathetic activity
		Regulates energy metabolism
		Improves insulin sensitivity and glucose tolerance
GLP1 agonists	Not all available in oral forms of administration	Cardio-reno-vascular risk reduction
Orlistat	Drugs interaction. Oily spotting of underclothes	Oral administration

RAAS, renin-angiotensin-aldosterone system; SGLT2, sodium-glucose transporter 2; GLP1, Glucagon-like peptide-1.

Non-surgical interventions have been useful in reducing urinary protein excretion in CKD. This was shown in a systematic review including different types of studies in patients with different causes of CKD (65); both observational and interventional protocols demonstrated a significant reduction in proteinuria, but no changes in GFR. For example, a 5-year lifestyle intervention obtaining a 5%–7% weight loss in prediabetics reduced the risk of T2DM by 58% compared with placebo (66).

However, diet and medical interventions may have some limitations in CKD because low carbohydrate diets are usually rich in proteins (67) and weight loss medications have not been adequately tested in established CKD (68).

Since weight reduction by conservative methods is difficult to succeed and maintain, with high rates of weight gain after an initial rapid loss (69), BS, also called metabolic surgery, was considered as an alternative with the hope of an easier, more effective, and sustained effect.

A large retrospective analysis of the 2010–2015 US National Inpatient Sample (NIS) database for the years 2010–2015 shows that the chance of developing CKD (adjusted for risk factors) and of reaching ESRD is lower in those with BS (56).

Indeed, BS is the most effective therapeutic option for morbid obesity and has been shown to induce modification of multiple risk factors involved in the progression of CKD (69). Moreover, BS improves albuminuria adjusted by the coexistence of diabetes, hypertension, age, race, and surgery technique (70).

## Bariatric surgery

There are many BS modalities, which are described later. The most frequently used has been the gastric bypass, but there is a trend to adopt the sleeve gastrectomy (SG) as the first choice (17) owing to its reduced complexity and comparable results with Roux-en-Y gastric bypass (RYGB) (71). All commonly performed procedures are done laparoscopically with a short hospital stay and each surgical technique has its own risks and complications.

### Gastric band

An adjustable silicone band is positioned around the top of the stomach after the esophagus to reduce its size as well as slow the passage of food through the restriction. With this surgical technique, approximately 30% of excess body weight is expected to be lost after 2 years of follow-up. Although this procedure is the one that people have most often heard of, it accounts for approximately 10% of bariatric procedures because it is deemed less effective than the others (72).

### Sleeve gastrectomy

In this case, the stomach is divided by stapling, which results in a ‘sleeve’-shaped stomach removing approximately 70% of it. Since parts of the stomach are removed, neurohormonal changes can be observed, such as decreased concentrations of Ghrelin, the hunger-stimulating hormone. This peptide is mainly produced by cells located in the gastric fundus, which is removed, and this can diminish appetite contributing to further weight loss. Approximately 60% of excess body weight is expected to be lost at 2 years after surgery (72).

### Gastric bypass

Two different types of gastric bypass are in use: the Roux-en-Y gastric bypass and the loop gastric bypass. In both techniques, a smaller stomach/pouch is made using staples. Then, a loop is shaped in the bowel, which forms a new way for the food to leave the stomach (shorter than before), which can subsequently cause some minor malabsorption contributing, together with the reduced volume of food, to an effective weight loss. Expected weight loss is approximately 60%–70% of the excess at 2 years post-surgery (72).

#### Beneficial effects of BS and their mechanisms

Surgery is considered a successful method to reduce appetite, and deciding which procedure is better for some people may be easy, but not for others. It is very effective in reducing weight, showing additional benefits in T2DM, high blood pressure, obstructive sleep apnea, and high cholesterol; other conditions, including osteoarthritis, skin conditions, and hormone-related problems such as polycystic ovary syndrome/subfertility, are likely to benefit, in addition to many others (73).

The first beneficial effect of BS was shown in diabetic patients (74) with a 66% of remission of DM with RYGB (28% with gastric band). In uncontrolled obese patients with diabetes (75), surgery (RYGB or SG) was effective to reduce BMI, glycated hemoglobin, antihypertensive and antidiabetic medications, and to improve the quality of life.

Following surgery, a weight loss of 25% can be maintained for the long term. The glucose-lowering effects rely on the effect of peripheral insulin resistance, but several interesting mechanisms, independent of weight loss, have been proposed as participants in the renoprotective outcome (17).

Indeed, evidence is growing that changes in gut hormones (such as GLP-1), intestinal microbiome, plasma bile acids, and nutrient-sensing mechanisms may participate in the long-term benefits (19, 76), although there is still scarce information on how weight loss may improve metabolic parameters in patients with CKD.

Metabolic surgery, by reducing increased intraabdominal pressure, improves renal vein pressure, increases renal blood flow, and normalizes GFR, plasma renin activity, and aldosterone levels, all of them involved in proteinuria (77). BS has been shown to reduce proteinuria and reverse hyperfiltration in obese people with conserved kidney function (78). In addition, in uncontrolled studies, BS improves other risk factors for CKD including cardiac disease, pulmonary hypertension, and sleep apnea (79–81).

Experimental data from animal models show that in parallel with improvements in weight and metabolic control, BS improved the kidney itself reducing fibrosis, inflammation, and oxidative stress at a transcriptomic level (82, 83).

A meta-analysis based on observational studies shows an improvement in albuminuria/creatininuria, both weight and blood pressure independently. With respect to GFR, the main demonstrated effect is a reduction in individuals with hyperfiltration and an increase in those with reduced eGFR (84).

A systematic review and meta-analysis of observational studies about the positive impact of BS on renal outcomes (84) showed reductions in albuminuria, proteinuria, and creatininemia in the overall group, and an improvement in GFR, especially in hyperfiltrating and CKD groups. Indeed, a reduction in filtration fraction is recognized as the main mechanism involved in the beneficial effect to improve renal damage in a subject who has lost weight (2). In addition, other studies focused on renal function showed that obese persons submitted to BS reduce in approximately 58% their decline of GFR by ≥30% and the risk of doubling serum creatinine at 5 years (85).

Changes in body surface area, after the surgery, induce problems in the interpretation of changes in measured GFR in individuals with preserved renal function (86) with the first report published in 1980 (87). They measured GFR with radioisotopic EDTA 12 months after intestinal bypass operation in patients with obesity observing a significant reduction in uncorrected GFR that disappeared after correction by body surface; the same was found measuring GFR with creatinine clearance. The best explanation for this fact is that knowing that the number of nephrons is established at birth, a reduction of hyperfiltration may be involved in the final GFR. A positive result on GFR, associated with a reduction in leptin levels, was observed in a small study of 13 patients with CKD (serum creatinine ≥ 1.3 mg/dL) measuring GFR with iothalamate (88). Attention must be paid to the marker used to evaluate renal function after BS because serum creatinine depends on the muscle mass, and its loss in the short term due to the surgical trauma may induce over-estimations of GFR evaluated with formulas only. In general, GFR equations combining serum creatinine and cystatin C have shown to be more accurate in estimating renal function, but there are also doubts regarding the validity of the adjustments for BMI in obese subjects, which may be imprecise (89). The direct measurement of GFR would be the ideal strategy (90, 91), but whether these changes in eGFR signify only hemodynamic changes or a true modification of the progression of CKD is still unclear (92).

The main effect of BS has been demonstrated in a reduction in the GFR of hyperfiltrating patients (65). The systematic review and meta-analysis of Bilha et al. (84) looking for the renal impact of BS in patients with obesity showed a significant decrease in serum creatinine in the general group, but not in patients with CKD; they could not find improvements in GFR (estimated or measured), except in CKD2, but a reduction in hyperfiltration was evident.

However, it may be more important to demonstrate if it may reduce the risk of developing CKD. A problem observed after the readout of the renal outcomes in the available published literature is that many of the manuscripts have shown short-term results. A systematic review (93) exploring the long-term results after SG suggests that BS can lead to considerable and lasting excess weight loss and significant modifications in obesity-related co-morbidities. In a prospective study, Friedman found that BS induced an improvement in the CKD risk categories in a large proportion of patients for up to 7 years, especially in those with moderate or high basal risk (94).

#### Effects of BS on risk factors of CKD

The impact of BS on risk factors of CKD was evidenced early after the introduction of the technique and is well described in a meta-analysis reported in 2014 (74). With respect to diabetes mellitus, BS induces remission of type 2 DM in 67% using RYGB and 29% with a gastric band (74) and is more effective than intensive medical therapy after 3 years of observation (75). A clear impact on renal protection in obese patients with type 2 DM has been evidenced, reducing albuminuria independently of the obtained changes in BMI, HbA1c, and systolic BP (95).

For hypertension, remission, defined as a blood pressure less than 140/90 without medications, was obtained in 38% of cases with gastric bypass (randomized controlled trial) and 17% with a gastric band (prospective cohorts). Confirming the positive effect, in adolescents, it induced remission of high blood pressure in 75% of subjects (96).

An improvement in dyslipidemia has also been found, with a remission rate of 60% after gastric bypass and 23% after the gastric band (74).

The impact of BS may be observed in patients without diabetes also; observational studies have shown a reduction in albuminuria and risk of ESKD after a follow-up of 18 years (97). In CKD, improvement in patients with obesity in the CKD stages has been observed in approximately half of the subjects, even in those at high risk at baseline (98).

In patients with moderate and high baseline CKD risk categories, BS is associated with an improvement in the CKD risk itself, in a large proportion of patients followed for up to 7 years. These findings support the introduction of CKD risk in presurgical evaluation for bariatric surgery (94). A retrospective cohort study of patients with CKD stage 3 or higher who received BS (RYGB or SG) showed slower declines in eGFR up to 3 years after surgery, which may be at least partly independent of weight loss (99).

#### Effects of BS on CKD progression

Metabolic surgery has been shown to reduce the likelihood of CKD progression and improve kidney function in observational studies (92), but there are few prospective reports of its effect on established CKD (47).

A recent retrospective study (56) of the US National Inpatient Sample database with 296,041 operated cases and 2,004,804 severely obese controls, found, even after adjusting for all CKD risk factors, 3.1 times higher incidence of CKD 3 and 1.13 times higher incidence of ESKD in the control group.

The CKD preventive effect of BS may be quantified from the data observed in the SOS study where a 47% of reduction in CKD stages 4 and 5 after a median 10 years follow-up was described. The protective effect was greater in patients with a baseline UACR > 34 mg/mmol, needing to treat 4 patients to prevent 1 case of CKD, stages 4 or 5 (100). Data from the United States have shown that patients with CKD and albuminuria moderate or severe have the best results, reducing the risk of progression to kidney failure by 70% at 2 years and 60% at 5 years (98).

Although in observational studies, surgery shows improvements in creatinine-based renal outcomes, long-term studies using other filtration markers less dependent on body mass are needed (86).

Renal protection induced by BS may last up to 9 years in patients with or without decreased baseline kidney function, hypertension, and diabetes (101). The best surgical modality to obtain the best renoprotection remains unknown, but although there are studies favoring RYGB, there are ongoing trials to solve this issue.

The results of randomized, controlled trials in patients with well-defined and staged CKD powered to detect surrogate endpoints of disease progression (102) or, even better, initiation of dialysis or mortality, such as NCT 04626323 (currently ongoing), is expected to solve the doubts.

## When to perform the surgery?

A meta-analysis of eight studies (six retrospective studies) including 766 patients with CKD of ≥3 described eGFR and/or creatinine improvement in 63% of the cases, independent of the surgery technique used, blood pressure, BMI, or presence of diabetes (84). Only one study revealed creatinine increase at a follow-up of 2 years.

Although it has been reported that individuals with a lower preoperative eGFR experience less weight loss after BS, others have found that preoperative renal function may not have an independent impact on postoperative weight loss in patients with eGFR of ≥30 mL/min (103). On the contrary, severe CKD (stages 4 and 5) may have the worst results in terms of weight loss, and some authors propose to perform the surgery at the earliest possible (104) because long-term studies suggest that surgical intervention might be the most beneficial at earlier stages of kidney disease (94).

More advanced stages of CKD do not appear to be statistically associated with an increased risk of early postoperative complications (105). Studies in the strict pre-dialysis phase are lacking, but it is possible that the post-surgery reduction of hyperfiltration and the post-operatory rapid catabolic state might accelerate the need for dialysis (106).

Paradoxically, in patients in dialysis, morbid obesity is protective if it is accompanied by muscle mass gain (107, 108). However, weight loss, if intentional, has not been demonstrated to be deleterious (109), and BS may be a good bridge to obtain a transplant (110) since the risk of surgical and post-surgical complications is increased in patients with obesity when transplanted, including retarded wound healing and infection, lymphocele, and CVD (111, 112). In patients in dialysis, before transplant, BS reduced mortality (regardless of developing ESRD) (113) incidence of diabetes and approximately 60% of cardiac disease (114).

Better selection criteria to submit patients to BS, which include the severity of obesity, goals (metabolic, effects on progression or regression of CKD), patients preferences, and tolerance to the risk, are also necessary (19).

## Obesity, bariatric surgery, and renal transplant

Obesity may impede access to kidney transplantation (19, 109, 115). In fact, it affects kidney transplant candidates, recipients, and potential living kidney donors deserving to be faced (116).

Meta-analysis has shown that patients with obesity after renal transplants have a higher risk of delayed graft function, risk of death, biopsy-proven acute rejection, and allograft loss (117). Patients who gained >15% of their initial weight during the first year showed higher mortality 10 years after, with a functioning kidney (118).

Some authors suggest that surgery should be considered 6–12 months after transplantation for patients with a BMI of >35 kg/m^2^ with cardiovascular and metabolic comorbidities and without previous bariatric procedures (116).

The intervention, whether performed before or after the transplant, is effective and safe, and the time to do so must be individualized for each patient (119).

The pharmacological effect of BS on immunosuppressive regimens must be tailored because potential changes in drug disposition after the anatomical and functional changes in the gastrointestinal tract (120) need to be faced with closer surveillance of drug blood levels.

## Complications of the surgery

Perisurgical complications are higher in patients having both obesity and renal disease (73, 121), and creatinine levels of >2 mg/dL have a higher risk of re-intervention, readmission, and acute kidney injury (122, 123). With a low incidence (<15% at 30 days), patients with CKD experience more frequent complications and readmissions after BS than patients without CKD, and it should not be a contraindication for the procedure (124) because the absolute risk is low and mortality does not increase (103, 125). Indeed, safety has improved substantially after the 2000s (73). Post-op acute kidney injury, if it occurs in a context without sepsis or in subjects who do not have previous CKD 4 or 5, has a good prognosis (126).

Long-term problems vary with the surgical technique used (127). Mechanical, stenotic problems may be observed after GS or RYGB. Gastroesophageal reflux is more typical of GS (128). RYGB has its own specific complications such as cholelithiasis (40% of the cases), incisional or internal hernias, dumping syndrome after ingestion of a high quantity of simple carbohydrates, and oxalate nephropathy. Reduced oral intake as well as altered stomach and small bowel absorption reduce the availability of various micronutrients, particularly iron, calcium, vitamin B12, thiamine, and folate (128) (Table 3).

**Table 3:** Non-surgical adverse effects of bariatric surgery.

Disorder	Mechanism	Clinical manifestations
Micronutrient (iron, calcium, zinc) and vitamin (Folate, B12, and Vit D) deficiencies	Exclusion of specific zone of absorption in duodenum Reduction in gastric acid Sub-clinical malabsorption	Iron-deficiency anemia
		Osteopenia and risk of fractures
Hyperoxaluria (mainly in RYGB)	Hyperoxaluria and hypocitraturia Malabsorption of fat, sequestration of calcium in soaps, increase of passive absorption of free oxalate in colon	Oxalate urolithiasis
		Oxalate nephropathy
Post operatory renal disfunction	Dehydration	Acute kidney injury
Changes in bioavailability of drugs	Changes in absorption of drugs	Changes in effect of medical therapies of comorbidities in chronic kidney disease
		Need for better surveillance of drug levels in transplant

RYGB, Roux-en-Y gastric bypass.

An important matter is that 1/3 of the patients submitted to BS may experience suboptimal results or significant weight regain in the first few postoperative years (129). Psychological aspects may be involved in the failure, since a high prevalence of pathologies, mainly eating and impulse control disorders can be found in patients requiring BS (129) that are not modified by the surgery (130), including the suicide risk (131). With any technique, failures are described, making lifelong follow-up necessary (128) to detect it and any potential complication at the earliest, performed by the best multidisciplinary BS team (132) available that should include at least a nutritionist, a psychologist, a gastroenterologist, and a nephrologist.

## Conclusion

An important issue in the therapy of obesity and its consequences is the persistence and duration of its effects. Although there is little doubt about the superiority of surgery over non-invasive therapies in terms of effectiveness, RCTs are expected to compare surgery treatment in addition to pharmacotherapy versus lifestyle changes and pharmacotherapy alone to help in deciding rationally the best recommendation in CKD, established or at risk of it. A synergic effect between surgery and medical therapies exists (17).

The most important point is an eventual change in the CV risk. Here, the best studies have been done using RYGB showing, at 7 years of follow-up, a reduction in adjusted mortality of 40%, mainly attributed to a reduction in diabetes-associated cardiovascular events (133). But these data must be analyzed with caution because patients who received surgery are probably healthier and more motivated than their severely obese controls. In addition, it must be highlighted that an increase in non-disease-related death (accidents or suicide) has been described after BS (133).

Then, the targeted use of metabolic surgery to improve the cardiovascular and renal risks must be balanced against the undesirable short and long-term risks and sequelae of the procedure, which can be of particular concern to patients with CKD (17).

The lack of randomized clinical trials, low follow-up rates, and poorly reported data regarding co-morbidities and quality of life in many of the studies indicate that these findings should be interpreted with caution.

The use of multitarget therapies with appropriate education and combinations of SGLT2 or GLP-1 agonists with RAS blockade and BS could obtain more integrated management of the inflammatory state and modulate adipocyte cytokines, in order to obtain the best renal protection (123).

Taking all of the above into account, if an increment in urinary albumin excretion is found in a patient with obesity, it is mandatory to intervene to reduce overweight, control hypertension, diabetes, and dyslipidemia with all the available tools to prevent future chronic renal damage (2).

## Author contributions

The author confirms being the sole contributor of this work and has approved it for publication.

## Funding

This work was supported by the Programa de Especialización en Medicina Interna, Facultad de Medicina, Universidad Austral de Chile.

## Conflict of interest

The author declares that the research was conducted in the absence of any commercial or financial relationships that could be construed as a potential conflict of interest.

## Publisher’s note

All claims expressed in this article are solely those of the authors and do not necessarily represent those of their affiliated organizations, or those of the publisher, the editors and the reviewers. Any product that may be evaluated in this article, or claim that may be made by its manufacturer, is not guaranteed or endorsed by the publisher.
